# Hyper-Null Models and Their Applications

**DOI:** 10.3390/e25101390

**Published:** 2023-09-28

**Authors:** Yujie Zeng, Bo Liu, Fang Zhou, Linyuan Lü

**Affiliations:** 1 Institute of Fundamental and Frontier Sciences, University of Electronic Science and Technology of China, Chengdu 610054, China; yujie_zeng@std.uestc.edu.cn (Y.Z.); zervel3@std.uestc.edu.cn (F.Z.); 2 Yangtze Delta Region Institute (Huzhou), University of Electronic Science and Technology of China, Huzhou 313001, China; 3 School of Cyber Science and Technology, University of Science and Technology of China, Hefei 230026, China

**Keywords:** null models, hypergraphs, randomness, network dynamics

## Abstract

Null models are crucial tools for investigating network topological structures. However, research on null models for higher-order networks is still relatively scarce. In this study, we introduce an innovative method to construct null models for hypergraphs, namely the hyperedge swapping-based method. By preserving certain network properties while altering others, we generate six hyper-null models with various orders and analyze their interrelationships. To validate our approach, we first employ hypergraph entropy to assess the randomness of these null models across four datasets. Furthermore, we examine the differences in important statistical properties between the various null models and the original networks. Lastly, we investigate the impact of hypergraph randomness on network dynamics using the proposed hyper-null models, focusing on dismantling and epidemic contagion. The findings show that our proposed hyper-null models are applicable to various scenarios. By introducing a comprehensive framework for generating and analyzing hyper-null models, this research opens up avenues for further exploration of the intricacies of network structures and their real-world implications.

## 1. Introduction

Over the past two decades, research on complex networks has made remarkable advancements and has become a fundamental paradigm applicable to sociology, biology, and other fields [1,2]. In the traditional domain of complex networks, the intermediary of interaction between nodes is typically represented by edges. Such networks are commonly referred to as pairwise networks. However, pairwise networks cannot describe interactions beyond two nodes, i.e., higher-order interactions, in the real world; for example, a metabolic reaction often involves a variety of compounds [3], and collaborative articles require contributions from multiple scholars [4]. Therefore, higher-order networks have been increasingly attracting the attention of scientific researchers [5,6]. Hypergraphs are one of the most common higher-order networks [7]. Unlike traditional graphs, where edges only link pairs of nodes, the edges of a hypergraph (called hyperedges) can connect more than two nodes [8]. Due to the flexibility of hypergraphs, the current related research mainly focuses on aspects like network dynamics on hypergraphs [7,9], the hypergraph neural network [10,11] and so on.

Topological statistics serve as a fundamental tool for describing complex network structures. However, due to the large variation in network sizes and the fact that the statistics are dimensionless, it is necessary to introduce a reference to compare the statistical properties of different networks [12,13,14]. The null model is a randomization-based model that can be generated by randomly rearranging the connections in the network [15,16]. The goal is to generate a network that changes some specific metrics while keeping other statistical metrics constant as the original network [17]. By comparing original networks with the null models, we can determine whether the structure or pattern in the original network is statistically significant and understand how altering the structure can affect the functionality.

Higher-order networks have a more complex structure compared to traditional binary networks [18], and their null models have been studied relatively little. Previous research on null models for hypergraphs, referred to as hyper-null models in this paper, has predominantly focused on specific configuration models [19], rather than exploring a comprehensive range of null models. Klimm et al. [20] proposed a new ER-hypergraph model. This is achieved by generating a random model through the selection of *K* nodes and assigning them to a hyperedge of size *K*. This method preserves the original network’s node number, hyperedge number, and hyperedge cardinality. Chodrow et al. [21] proposed two new configuration models, which keep the hyperdegree and hyperedge size constant, respectively, i.e., the vertex-labeled configuration model and the stub-labeled configuration model. The construction of these two models involves sampling specific attributes from the original network using a Markov chain Monte Carlo scheme. Miyashita et al. [22] proposed random hypergraph models, preserving the pairwise joint degree distribution and the clustering coefficient by defining a new property: a distance for which the edges can be rewiring if the distance is decreasing.Nevertheless, these methods fail to provide a comprehensive definition for a range of hyper-null models. Instead, they only focus on specific configuration models that address randomness from a limited perspective. Therefore, a more all-encompassing set of null models is needed in order to fully capture and analyze the randomness and structural characteristics of hypergraphs. In 2021, Nakajima et al. [23] introduced the dK-series null models for a hypergraph based on the configuration model. It can maintain some statistical characteristics of the original network. However, both the ER graph and configuration model generate the null models by starting with an empty network. These methods involve reconnecting all the hyperedges and excluding some errors (self-loops and repeated hyperedges), which can lead to increased complexity or potential loss of other important network features.

Another gap of hyper-null models lies in the fact that some models juxtapose the retained feature relationships without exhibiting any inclusion relation, making randomness difficult to distinguish. For example, the null models that displace the preservation of the hyperedge degree and the preservation of the nodes’ hyperdegree both belong to the 1k null model, and the two are juxtaposed. Therefore, it is imperative to adopt metrics that measure the randomness of the hypergraph to aid in understanding the relationship between the hyper-null models.When researchers investigate the randomness of networks, entropy is commonly employed as a quantification method [24]. Among the established methods are Shannon entropy [25], degree distribution entropy [26], and others. Networks with higher entropy values generally exhibit greater randomness and uncertainty, while networks with lower entropy values often display more orderliness and predictability [25]. By comparing the entropy values of different networks, we gain insights into their unique characteristics and evolution processes [27]. At present, some scholars have extended entropy from pairwise networks to higher-order networks. Hu et al. [28] employed the node degree distribution to estimate the hypergraph entropy by fitting it to the Shannon entropy formula.

In this paper, we extend the concept of the null model from pairwise networks to hypergraphs and present a novel approach to constructing hyper-null models, namely the hyperedge swapping-based method. Additionally, we employ Shannon entropy as a key metric to evaluate the randomness of the six types of hyper-null models across four datasets. Moreover, we propose a framework to analyze their comparative results by applying the hyper-null models and the original network in the same case. And it is validated in the analysis of topological statistics, hypergraph dismantling and epidemic contagion.

Our contributions are three-fold as follows:

- We propose a new method to construct null models for hypergraphs and summarize the relationship between our proposed hypergraph models.
- We introduce the concept of entropy to quantify the randomness of networks, retaining different statistical properties, and explore the relationship between randomness and the network structure.
- We utilize topological statistics analysis, network dismantling, and epidemic contagion to showcase the universality of the framework employed in the original network and its hyper-null models.

The paper’s structure unfolds as follows: Section 2 lays the groundwork by introducing the methods used in this study. This includes explaining the fundamental aspects of hypergraphs, clarifying the concept of randomness, and providing an in-depth presentation of the novel hyper-null model method devised in this research. Section 3 validates the effectiveness of the proposed method. It not only scrutinizes the method’s performance but also delves into the intricate interplay between randomness and the dynamics of networks through the lenses of hypergraph epidemic contagion and hypergraph dismantling. Finally, Section 4 provides a comprehensive summary of this paper.

## 2. Methods

### 2.1. Hypergraphs

The hypergraph is a kind of higher-order network which mainly focuses on interactions beyond pairwise connections. A hypergraph H=(V,E) contains a node set $V=\{v_{i}:i\in \left[1,n\right]\}$ and a hyperedge set $E=\{e_{j}:j\in \left[1,m\right]\}$, where each hyperedge is a non-empty subset of *V* such that ${⋃}_{j\in \left[1,m\right]}e_{j}=V$ [29]. Here, *n* denotes the number of nodes, and *m* denotes the number of hyperedges.

### 2.2. Hyperedges and Hypertriangles

**Figure 1:**
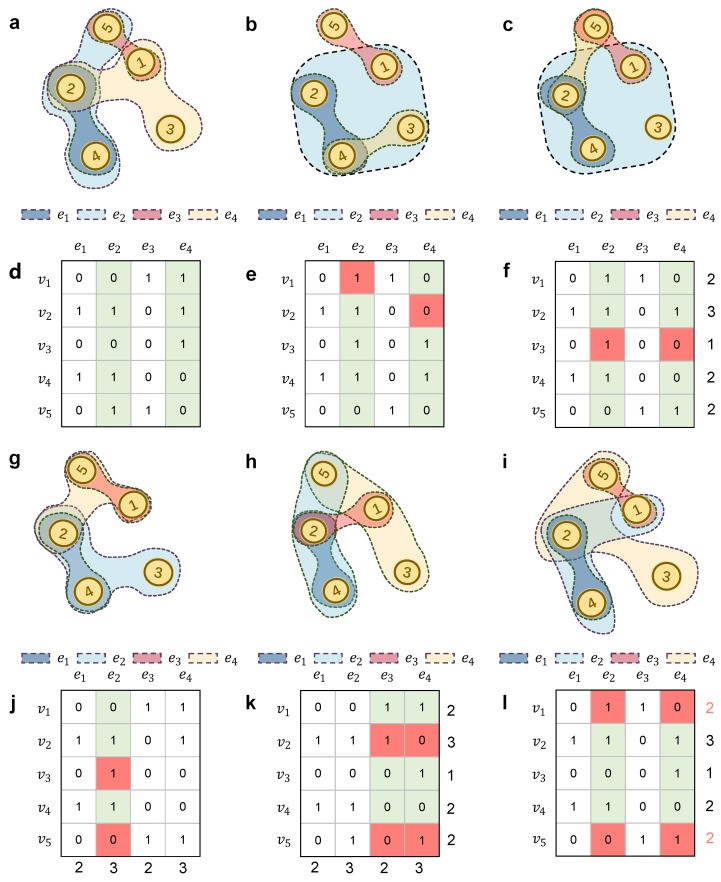
A hypergraph and its hyper-null models. (**a**,**d**) denote the original hypergraph and its incidence matrix, (**b**,**e**) represent the H0k null model and its incidence matrix, (**c**,**f**) denote the H1k-HD null model and its incidence matrix, (**g**,**j**) denote the H1k-HED null model and its incidence matrix, (**h**,**k**) denote the HD-HED null model and its incidence matrix, (**i**,**l**) denote the H2k null model and its incidence matrix. The green columns represent the randomly chosen hyperedges, while the red boxes denote the nodes that undergo a single swap during the transformation process.

The hyperedge set is the basic component of a hypergraph. A hyperedge is a subset of the node set. A hypertriangle is defined as a sequence consisting of three different nodes and three different hyperedges [30]. For example, the nodes $v_{1},v_{2},v_{5}$ and their corresponding hyperedges $e_{3},e_{4},e_{2}$ form a hypertriangle in Figure 1a. Notice that the nodes $v_{1},v_{2},v_{3}$ cannot form a hypertriangle in Figure 1a, because they are in the same hyperedge $e_{4}$.

### 2.3. Statistics of Hypergraphs

Statistics can be used to measure some topological properties of networks. For example, in pairwise graphs, the node degree—representing the number of neighboring nodes—is a measure of the importance of a node. Similarly, hypergraphs also have their own set of metrics. Here are some fundamental ones.

**Hyperdegree**: The hyperdegree refers to the node degree in hypergraphs. It represents the number of hyperedges that a node is located in.

**Hyperedge degree**: The hyperedge degree denotes the number of nodes that are contained in a hyperedge.

**Co-average hyperdegree**: The co-average hyperdegree of node *i* represents the average hyperdegree of node *i*’s neighbors.

**Hyperdegree distribution**: The hyperdegree distribution denotes the probability that a randomly chosen node across the entire hypergraph will have a hyperdegree of *k*.

**Joint hyperdegree distribution**: The joint hyperdegree distribution denotes the distribution of hyperdegrees among nodes within each hyperedge.

**Hypergraph clustering coefficient**: The clustering coefficient of a node represents the ratio of the number of existing hyperedges between its neighbors to the number of all possible hyperedges. The clustering coefficient of the hypergraph is the average value of the clustering coefficients of all nodes in the hypergraph.

**Average neighbor degree**: The average neighbor degree of a node denotes the average of the neighbors’ degree of it.

**Strength**: The strength of node *i* represents the total number of hyperedges that node *i* shares with any other node in the hypergraph.

**Assortativity**: Based on the assortativity *r* proposed by Newman [31], we calculate the assortativity for hypergraphs as

(1) $$r=\frac{M^{-1}\sum_{i}j_{i}k_{i}-{\left[M^{-1}\sum_{i}\left(j_{i}+k_{i}\right)\right]}^{2}}{M^{-1}\sum_{i}\frac{1}{2}\left(j_{i}^{2}+k_{i}^{2}\right)-{\left[M^{-1}\sum_{i}\frac{1}{2}\left(j_{i}+k_{i}\right)\right]}^{2}},$$

where *M* denotes the number of edges, and $j_{i}$ and $k_{i}$ are the degrees of the nodes at the ends of the *i*-th edge, where i=1⋯M.

### 2.4. Matrices of Hypergraphs

#### 2.4.1. Hyperdegree Matrices

The hyperdegree matrix *D* of a hypergraph denotes the number of hyperedges in which the node is located. It can be expressed as

$$D_{n\times n}=\left(d_{ij}\right),$$

where $d_{ij}$ represents the number of hyperedges that contain both node *i* and *j*. Further, $d_{ii}$ is the hyperdegree of node *i*.

#### 2.4.2. Hyperedge degree Matrices

The hyperedge degree matrix $D_{e}$ of a hypergraph represents the size of each hyperedge, that is

$$D_{em\times m}=\left(d_{eij}\right),$$

where $d_{eij}$ denotes the number of nodes in both hyperedge *i* and *j*. Moreover, $d_{eii}$ means the hyperedge degree of hyperedge *i*.

#### 2.4.3. Incidence Matrices

The incidence matrix *C* of a hypergraph represents the relationship between nodes and hyperedges. It can be expressed as

(2) $$C_{n\times m}=\left(c_{ij}\right).$$

For each item $c_{ij}$ in the incidence matrix of *C*, if hyperedge $e_{j}$ contains node *i*, then $c_{ij}=1$; else, $c_{ij}=0$. Because a hyperedge can contain more than two nodes, a single column in an incidence matrix can contain multiple instances of “1". The incidence matrix of the toy hypergraph in Figure 1a is shown in Figure 1d.

#### 2.4.4. Adjacency Matrices

The adjacency matrix *A* of a hypergraph represents the connection relationship between nodes. It can be expressed as

(3) $$A_{n\times n}=\left(a_{ij}\right).$$

In a hypergraph H, the item $a_{ij}$ of the adjacency matrix denotes the number of hyperedges that contain both node *i* and node *j*. As two nodes can be contained in more than one hyperedge, $a_{ij}$ is usually an integer greater than or equal to 0.

The adjacency matrix can also be represented by the incidence matrix

(4) $$A=CC^{T}-D,$$

where *D* is the hyperdegree matrix of H. Note that the diagonal elements are zero in the adjacency matrix. The sum of the *i*-th row in *A* denotes the strength of node *i*. Moreover, the hyperedge adjacency matrix is defined as

(5) $$B_{m\times m}=\left(b_{ij}\right)=C^{T}C-D_{e},$$

where $b_{ij}$ represents the number of nodes shared by two hyperedges, and $D_{e}$ represents the degree matrix of hyperedges, which is a diagonal matrix, and each entry on the diagonal is a hyperedge degree.

### 2.5. Randomness

In this paper, we use the hypergraph entropy proposed by Hu et al. [28] to quantify the randomness of hyper-null models. This method uses the node degree distribution to fit the Shannon entropy formula. Specifically, given the node hyperdegree distribution $d_{1},d_{2},\cdots ,d_{n}$, the hypergraph entropy can be calculated as

(6) $$I\left(H\right)=-\sum_{i=1}^{n}\left(\frac{d_{i}^{t}}{\sum_{j=1}^{n}d_{j}^{t}}\log\frac{d_{i}^{t}}{\sum_{j=1}^{n}d_{j}^{t}}\right)$$

### 2.6. Hyper-Null Models Based on Hyperedge Swapping

In order to generate the hyper-null models more accurately and efficiently, in this section, we present methods for constructing six types of hyper-null models based on hyperedge swapping. The construction rule for hypergraphs is an extension of the rule used in pairwise networks [15]. The original toy hypergraph is shown in Figure 1a. Its hyper-null models with different orders, which are obtained by changing the incidence matrix of the original hypergraph, are shown in Figure 1b,c,g,h,i. The detailed process of generating each type of hyper-null model is described below. In our experiment, the number of repetitions is 10 times the number of hyperedges.

#### 2.6.1. Hyper-0k Null Model

The hyper-0k null model (H0k null model) maintains the same number of nodes and hyperedges as the original hypergraph, thus ensuring that the average hyperdegree of the original hypergraph is preserved in the null model. To construct the null model of the toy hypergraph (Figure 1a), we need to arrange five nodes among four hyperedges, where each node is in more than one hyperedge, and each hyperedge contains at least one node. The construction process involves randomly selecting two columns in the incidence matrix (corresponding to two hyperedges in the network), then randomly choosing an entry from each of these columns (corresponding to one node in each hyperedge), and finally swapping these two selected entries if they have distinct values. As illustrated in Figure 1b,e, we randomly select two columns in the incidence matrix, say, $e_{2}$ and $e_{4}$, and then swap the “0” and “1” entries corresponding to these two columns.

#### 2.6.2. Hyper-1k Null Model with Hyperdegree Constant

The hyper-1k null model with hyperdegree constant (H1k-HD null model) is extended from the 1k null model in pairwise networks and keeps the hyperdegree distribution constant. As shown in Figure 1c,f, to construct the H1k-HD null model, we begin by randomly selecting two hyperedges, $e_{2}$ and $e_{4}$, which are the second and fourth columns of the incidence matrix in Figure 1f. Then we choose two entries that are from these two columns and are located in the same row (corresponding to one node in the two selected hyperedges). If these two entries are different, we swap them. This process aims to preserve the hyperdegree of nodes in these two selected hyperedges, which corresponds to the sum of each row in the incidence matrix. In Figure 1f, the red boxes corresponding to node $v_{3}$ in $e_{2}$ and $e_{4}$ are swapped. This swap ensures that the row sum of the matrix remains constant before and after the exchange. Repeat this process several times to construct the H1k null model.

#### 2.6.3. Hyper-1k Null Model with Hyperedge Degree Constant

The hyperedge degree null model with hyperedge degree constant (H1k-HED null model) is also extended from the hyper-1k null model and keeps the hyperedge degree constant. As shown in Figure 1g,j, to construct an H1k-HED null model, we randomly select a hyperedge $e_{2}$, which is the second column of the incidence matrix in Figure 1j. Then we randomly choose two entries with different values in this column (corresponding to two nodes in this hyperedge) and swap them. The aim of this process is to preserve the sum of each column in the incidence matrix. In Figure 1j, the red boxes corresponding to nodes $v_{3}$ and $v_{5}$ in $e_{2}$ are swapped. The column sum of the matrix remains constant before and after the exchange. Repeat this process multiple times to create the H1k-HED null model.

#### 2.6.4. Hyperdegree–Hyperedge Degree Null Model

The hyperdegree–hyperedge degree null model (HD-HED null model) keeps the hyperdegree and hyperedge degree constant. As shown in Figure 1h,k, to construct an HD-HED null model, we randomly select two hyperedges $e_{3}$ and $e_{4}$, which are the third and fourth columns of the incidence matrix in Figure 1k. Then we pick two pairs of entries from these hyperedges, ensuring that each pair is from the same row while containing distinct entry values. Then we compare the selected two pairs: if the two entry values in one pair differ from their counterparts in the other pair, we swap the two pairs. This process preserves the sum of each row and column of the incidence matrix. In Figure 1k, the red box pairs in the second and fifth rows denote the two entry pairs that are swapped. That means that $v_{2}$ is put into $e_{3}$ and $v_{5}$ is put into $e_{4}$. The HD-HED null model can be constructed by repeating the process several times.

#### 2.6.5. Hyper-2k Null Model

The hyper-2k null model (H2k null model) keeps the joint hyperdegree distribution constant. This constraint requires the H2k null model to maintain a constant hyperdegree sum of nodes within each original hyperedge. As shown in Figure 1i,l, to construct an H2k null model, we first pick two hyperedges randomly, say, $e_{2}$ and $e_{4}$ here. Then we pick one node at random in each of the two hyperedges, which is $v_{1}$ and $v_{5}$. Note that the node which is common to both the two hyperedges cannot be selected. If these two nodes have the same hyperdegree, they can be swapped. For example, the red boxes in Figure 1l indicate that $v_{1}$ and $v_{5}$ can be swapped because they have the same row sum in the incidence matrix. Repeating this process several times leads to the construction of the H2k null model.

#### 2.6.6. Hyper-2.25k Null Model

The hyper-2.25k null model (H2.25k null model) keeps the joint hyperdegree distribution and clustering coefficient constant. To construct a hyper-2.25k null model, we need to calculate the clustering coefficient of the network after the same node selection operation in the H2k null model construction. If the clustering coefficient remains unchanged, the exchange can be performed; otherwise, we need to find other two nodes that satisfy the conditions. Repeating this process several times leads to the construction of the H2.25k null model.

It should be noted that, across all orders of null models, an empty hyperedge is not permissible. So, it is necessary to check it after each exchange operation; if the hyperedge is not empty, the exchange is successful.

**Figure 2:**
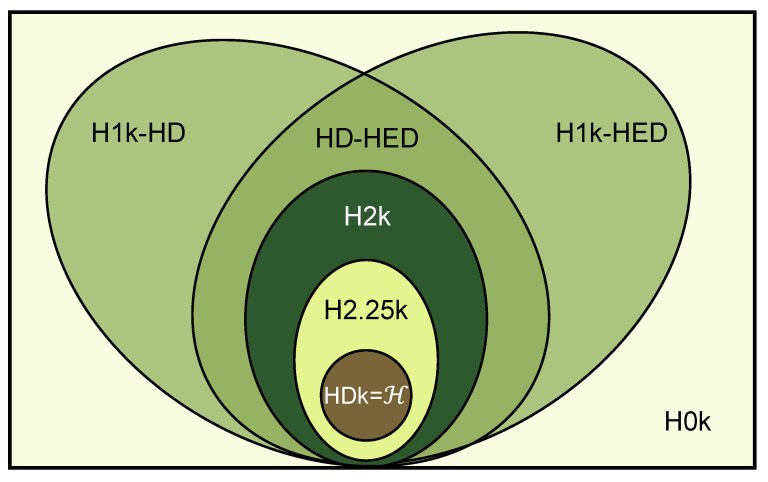
The relationship between hyper-null models. From the inside to the outside are the null models of different orders. HDk=H denotes the original network.

We describe the relationship between the above hyper-null models in Figure 2 and generalize this relationship as H0K⊆H1k−HD/H1k−HED⊆H2k⊆H2.25k⊆HDk=H. The size of the null model’s area indicates its level of randomness: a larger area represents stronger randomness, while a smaller area represents weaker randomness. The closer the occupied area is to the original network, i.e., the core part, the more features it retains from the original network. As depicted in the figure, the H0K null model demonstrates the strongest randomness and retains the fewest features from the original network. It is important to note that both the H1k-HD and the H1k-HED belong to the 1k null model, but they are distinct from each other. They do not have a strict theoretical inclusion relationship. On the other hand, the HD-HED null model simultaneously maintains the properties of both H1k-HD and H1k-HED. Previous studies only investigated statistical properties or dynamical processes within the original network itself. Yet here, by using our proposed hyper-null models, we are able to use a framework that comparatively analyzes these properties in hypergraphs, which provides a comparable quantitative result for us to analyze the networks with different scales. And the impact of different network properties or randomness on application scenarios can also be summarized.

## 3. Results

In this section, we discuss the relationship between the randomness of the network and its structure and function. The analysis begins by examining the variations in network structure across different levels of randomness. Subsequently, the impact of network randomness on network function is evaluated through dismantling experiments and epidemic contagion experiments. Furthermore, this study investigates the suitability of different null models as benchmark models for evaluating network properties.

### 3.1. Data Description

Four different datasets are used in this experiment: Algebra [32], Bars-Rev [32], iAF1260b [33], and iJO1366 [33]. These datasets exhibit varied topological features, allowing us to assess the robustness of our proposed method across diverse network structures. The statistical characteristics are shown in Table 1.

- **Algebra**: A question–answer network, which is collected from MathOverflow.net, where the nodes denote users, and the users who answered the same question are enclosed in a hyperedge.
- **Bars-Rev**: A review hypergraph collected from Yelp.com, where a hyperedge consists of the users who reviewed the same bars.
- **iAF1260b and iJO1366**: The metabolic hypergraph where nodes denote metabolites and the hyperedges represent the reaction that is involved in the same metabolic.

**Figure 3:**
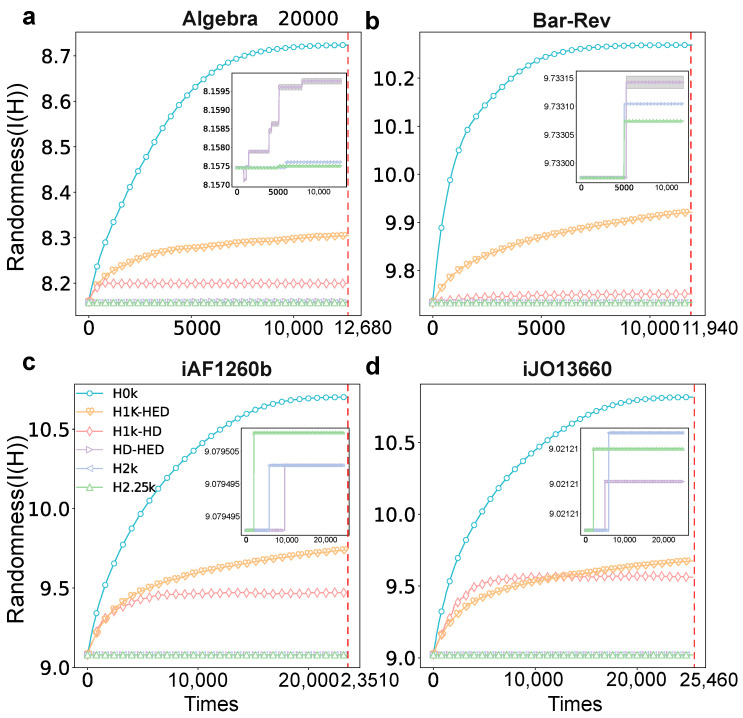
The trend of randomness on four datasets. (**a**–**d**) demonstrate how the randomness of different null models changes as the times of swapped hyperedges increase in datasets Algebra, Bar-Rev, iAF1260b and iJO13660. The x-axis denotes the times of hyperedge swapping, while the y-axis represents the randomness (degree distribution entropy) of the network. The subfigures in the top right of a, b, c, d are the enlarged drawing of HD-HED, H2k and H2.25k. The red dotted line denotes actual swapped times. Here, we choose 10 times the number of hyperedges.

Initially, we aim to verify the validity of our null model generation by quantifying the randomness exhibited by various types of null models. This crucial step involves generating multiple instances of null models with different orders and assessing their level of randomness. The experiments involve generating null models of various orders multiple times (specifically, 10 times) and measuring the average randomness of these models as the times of hyperedge exchanges increase. The results of these experiments are depicted in Figure 3. Figure 3 showcases the progressive increase in randomness observed in null models of varying orders as the times of hyperedge swaps escalate. Upon reaching a certain threshold of exchanges, the model’s randomness enters a stationary phase. Furthermore, through a comparative analysis of the randomness across different null models, we observe a gradual reduction in randomness as the order of the null model increases. It can be observed in Figure 3 that there is much difference between H0k, H1k-HD and the original network, and the HD-HED null model also exhibits some differences from the original network in some hyperedges. Yet, the H2k and H2.25k null models demonstrate relatively closer resemblance to the original network as a whole.

### 3.2. Statistical Analysis Based on Hyper-Null Models

**Figure 4:**
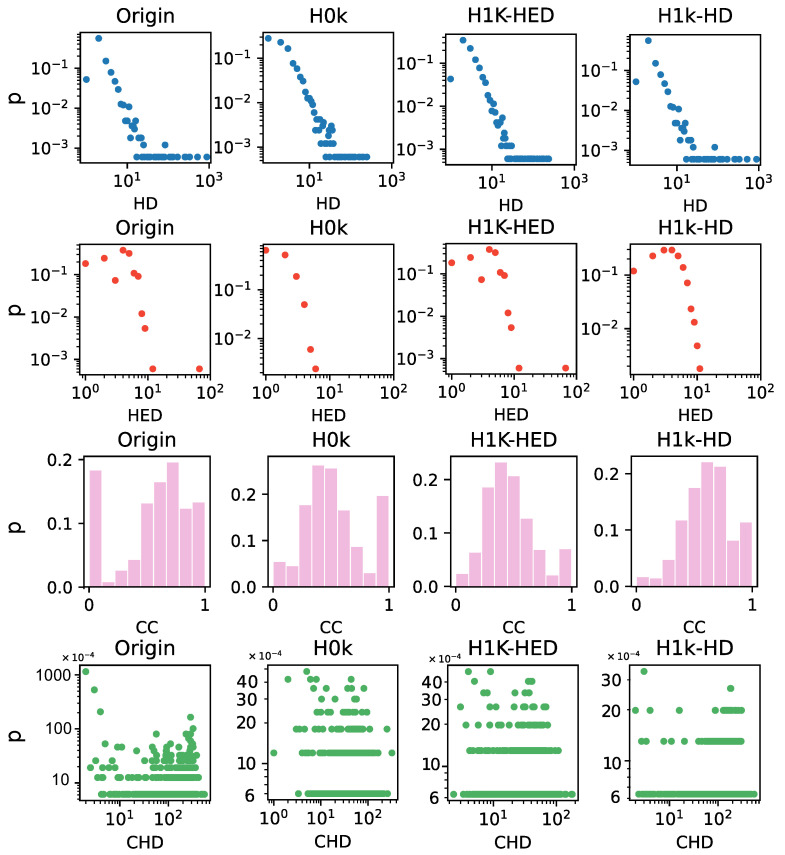
Statistical indices of network iAF1260b and its hull models. The x-axis in four rows from top to bottom represents the distribution of hyperdegree (HD), hyperedge degree (HED), clustering coefficient (CC) and co-average hyperdegree (CHD). The y-axis of each subgraph represents the proportion *p* of all nodes that are under the current value.

We proceed to investigate the topological properties of the networks with several metrics. Specifically, we calculate four fundamental statistical indices, namely hyperdegree, hyperedge degree, clustering coefficient, and co-average hyperdegree, for the iAF1260b network in Figure 4. The statistical indices for the other three datasets are provided in the Supplementary Material. Since the distributions of HD-HED, H2k, and H2.25k in these four metrics are basically consistent with the original network, they are not shown here.

We observe that, with the exception of the H0k and the H1k-HED null models, all other null models preserve the same hyperdegree distribution as the original network. This aligns with the rules followed when generating different null models. Additionally, the basic null model attribute, that is, having the same hyperedge degree as the original network, is also observed in the H1k-HED, HD-HED, H2k and H2.25k null models, which ensures a constant hyperedge degree. In the H0k null model, we observe a higher number of nodes with small hyperdegrees compared to other null models and the original network. As the exchange proceeds, the number of nodes with large hyperdegrees decreases gradually. This significant number of nodes with uniform hyperdegree contributes to the network’s robustness and facilitates information transmission. Similarly, nodes in the H0k null model exhibit relatively uniform co-average degrees. This characteristic also facilitates the dissemination of information or the spread of diseases within the network.

The hyperedge degree distribution of these null models exhibits a decreasing trend as the hyperedge degree increases, while the number of hyperedges with large hyperedge degrees in H0k is the smallest. This loose structure in the network corresponds to a lower likelihood of network collapse. Regarding the clustering coefficient, the number of nodes with a clustering coefficient of around 0.5 in H0k is the highest. The H0k null model has the lowest clustering coefficient, followed by the H1k-HED null model, and then the H1k-HD null model. The original network has the highest clustering coefficient among all models. This observation suggests that there are dispersion and uniform nodes in the H0k and other order null models compared to the original network. These relationships make the network more resistant to dismantling.

In addition to providing the distribution of the local indicators, we also analyze the important global statistics of these four empirical networks as shown in Table 2.

Table 2 displays the assortativity of the original networks in the first row. It can be observed that Algebra is more neutral, while Bars-Rev tends to be assortative, and the iAF1260b and iJO1366 networks tend to be disassortative. To analyze the significance, we introduce a null model-based analytical framework through introducing the significance metric μ, which is calculated as the ratio of the metric value of the current model and the original network. We compute the assortativity of six types of null models and their significance metric μ in comparison to the original network. As the randomness of the null model increases, the assortativity tends to be neutral. The H0K null model has the strongest randomness, and its performance is significantly different from the original network in all four empirical networks. The higher-order null models, like the HD-HED, H2K, and H2.25K null models, show assortativity that is relatively closer to the original networks. Hence, we choose the two hyper-1K null models for our analysis here. By comparing the deviation of μ and 1, we can analyze the significance of the assortativity of a network. As observed in the table, the μ of the assortativity of Bars-Rev is better than that of Algebra, iAF1260b and iJO1366, which means that the assortativity of Bars-Rev is more significant than the other three datasets. As for the clustering coefficient, the μ of Bars-Rev is less than that of Algebra, iAF1260b and iJO1366, which means that the clustering coefficient of Algebra, iAF1260b and iJO1366 is more significant than that of Bars-Rev. The average neighbor degree of Algebra and Bars-Rev is more significant because the μ of these two datasets is larger than that of other datasets.

In summary, as the order of the null model increases, that is, as the randomness increases, the structure becomes closer and closer to the original network. And the network with moderate randomness (such as H1k-HD and H1k-HED) is more suitable as the benchmark of the network.

### 3.3. Hypergraph Dismantling

In the following two sections, we analyze the impact of network randomness on network function from the perspective of dynamics, specifically network dismantling and network epidemic contagion. For network dismantling, we dismantle the hypergraph in two ways: removing nodes and removing hyperedges.

#### 3.3.1. Hypergraph Dismantling by Removing Nodes

**Figure 5:**
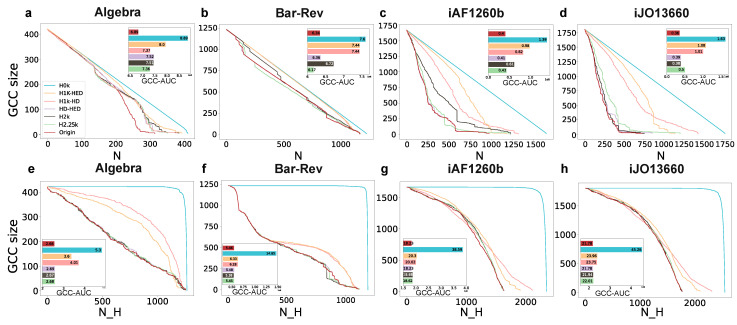
Dismantling networks by removing nodes (**a**–**d**) and hyperedges (**e**–**h**). The x-axis denotes the number of removed nodes (**a**–**d**) or hyperedges (**e**–**h**). *N* denotes the number of removed nodes while $N_{H}$ represents the number of removed hyperedges. The y-axis represents the GCC size of the network. The small panels inside (**a**–**h**) denote the GCC-AUC of each null model in four datasets. The GCC-AUC of each null model is shown at the right end of the column.

This approach involves iteratively removing one node at a time and calculating the size of the giant connected component (GCC) in the remaining network. The process continues until the GCC size falls below the target size. The evaluation metric used is the number of nodes removed, with a smaller value indicating a better outcome. In this experiment, the target GCC size is set to 0.01×N, and the list of removed nodes can be sorted using a hyperdegree, that is, nodes with larger hyperdegree will be preferentially removed. As shown in the upper row of Figure 5, the dismantling trend of the original network and various orders of null models by removing nodes according to the hyperdegree are displayed. The inset graph illustrates the corresponding area under the GCC curve (GCC-AUC, AUC for short) of each line of the number of removed nodes (hyperedges) versus the GCC size of the network. The calculation of AUC comprises summing the GCC values for each curve at each step until the GCC size is 0.01. The larger the AUC, the more slowly GCC decreases when removing nodes, and the more cost is required for dismantling. On the other hand, the network is easier to unravel. The result of the number of removed nodes of each model is shown in Supplementary Materials.

It reveals that both the AUC and the number of removed nodes in the original network are the lowest among the four datasets. So, it can be concluded that as the randomness decreases in these seven networks (the original network and its corresponding six hyper-null models), the network experiences a more rapid dismantling process.

#### 3.3.2. Hypergraph Dismantling by Removing Hyperedges

Peng et al. [34] propose a dismantling method by removing hyperedges based on the hyperedge degree. This process involves iteratively removing a hyperedge and calculating the GCC size of the remaining network until the GCC size reaches the target size, which is 0.01×N in this experiment. Figure 5 demonstrates that the rate of network collapse increases as the randomness decreases in these seven networks (the original network and its corresponding six hyper-null models). This observation further confirms the inverse relationship between dismantling speed and network randomness. Additionally, the initial leveling off followed by a rapid and steep drop in H0k can be attributed to the presence of hyperedges with large hyperedge degrees in the network.

### 3.4. Hypergraph Epidemic Contagion

To study the spreading performance of different order null models and explore the impact of the randomness of different order null models, we employ the SIR epidemic spreading model on hypergraphs [35] to achieve this goal. In the SIR epidemic spreading model, each node can have one of three states, susceptible (S), infectious (I), and recovery (R) state at a time step. Susceptible nodes have the potential to be infected with a probability of β, infectious nodes have the probability of γ to recover, and nodes that have recovered acquire immunity and will not be infected again. At the beginning of the experiment, all nodes are set in the susceptible state, and a fraction of nodes are chosen as seeds, with their states set as infectious. Within the epidemic spreading process, the infection probability $\beta_{i}$ of each node *i* is not the same, and needs to be individually calculated. Specifically, for each hyperedge e∈E containing node *i*, if the number of infectious nodes in *e* exceeds θ, then this hyperedge itself becomes infectious. Any nodes infected by this hyperedge will be included in the overall infection node number $n_{all}$. Therefore, the infection probability $\beta_{i}$ of node *i* can be calculated as $\beta_{i}=1-e^{-\beta \times n_{all}}$. In this experiment, we set θ=5, γ=1. The initial size of the seeds is 0.01×N, and the propagation capability is measured based on the number of recovery state nodes in the steady state. We repeat this experiment 100 times to decrease the impact of noise and randomness.

**Figure 6:**
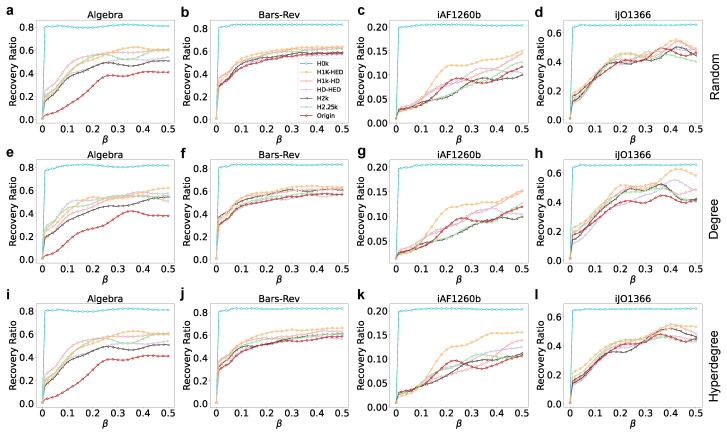
SIR epidemic contagion across four datasets with different infection rates. The first (**a**–**d**), second (**e**–**h**), and third (**i**–**l**) rows denote the epidemic results for 1% initial infected nodes selected randomly, based on degree, and using hyperdegree, respectively. The x-axis represents the different infection rates β and the y-axis denotes the recovery number ratio at the steady state.

Figure 6 shows that with the increment of the order of the null model, the final epidemic spreading capacity shows a downward trend. That is, the randomness of the network is negatively correlated with the epidemic-spreading ability. We also conduct supplementary experiments using the same parameter settings, except for varying the initial seed size, which is set to 0.02×N and 0.05×N (the results are shown in Supplementary Materials Figures S4 and S5). To explore the propagation capabilities of different networks, we study how the infection probability β (0.1, 0.3, 0.5) influences the spreading capacity of seeds in the four aforementioned datasets. We select the top 0.01×N nodes ranked by the hyperdegree. The result is presented in Table 3, Tables S2 and S3 (Tables S2 and S3 are presented in Supplementary Materials).

Table 3, Tables S2 and S3 show that there is no big difference in the propagation results between the original network and H2k and H2.25k. However, the propagation ability of the null model of other orders decreases as the order increases.

## 4. Conclusions

This paper introduces a novel approach to constructing null models in hypergraphs. The order of the null model corresponds to the level of similarity it retains with the original network, thereby establishing a relationship between the order of null models and the degree of randomness. Furthermore, the hyper-null model we propose can adequately provide references for the original network, i.e., it constitutes a research framework that allows a comparative analysis of the original network and hyper-null models. Additionally, we analyze the randomness of the four empirical networks and their null models, applying the framework based on hyper-null models to the analysis of the four empirical networks concerning three aspects: statistical properties, dismantling, and epidemic contagion.

We analyze some important local and global statistics. We find that the biggest advantage of using hyper-null model analysis is the ability to give a quantitative evaluation of the significance of the statistical properties of networks with different sizes, compared to computing only the dimensionless statistics of the original network, that is, to quantify the degree of nontriviality of the statistics themselves. Network dismantling experiments demonstrate that the resilience of a network is positively correlated with its degree of randomness. Similarly, epidemic contagion experiments reveal that the network’s ability to facilitate the spread of contagion is also influenced by its level of randomness.

The hyper-null model proposed in this paper is constructed based on the method of local hyperedge disruption, which is simple to operate and fully preserves the essential features of the original network. The universality of the analysis framework based on the hyper-null models is demonstrated through different applications. And it can provide a formidable tool in the analysis of hypergraphs for complex networks and interdisciplinary researchers. 

## Figures and Tables

**Table 1:** Basic characteristics of the studied networks.

Networks	*n*	*e*	$\langle k\rangle$	$\langle k_{H}\rangle$	*C*	Node	Hyperedge
Algebra	423	1268	78.90	19.53	0.79	User	Question
Bars-Rev	1234	1194	174.30	9.62	0.58	User	Bar review
iAF1260b	1668	2351	13.26	5.46	0.55	Metabolite	Metabolic interaction
iJO1366	1805	2546	16.92	5.55	0.58	Metabolite	Metabolic interaction

Here, *n* denotes the number of nodes; *e* denotes the number of hyperedges; $\langle k\rangle$ represents the average degree; $\langle k_{H}\rangle$ denotes the average hyperdegree; and *C* represents the average clustering coefficient.

**Table 2:** The assortativity, clustering coefficient, average neighbor degree and their μ of each network.

Statistics	Network	Original	H0k	H1k-HED	H1k-HD	HD-HED	H2k	H2.5k
Assortativity (μ)	Algebra	−0.10	−0.02 (0.20)	−0.03 (0.30)	−0.05 (0.50)	−0.08 (0.80)	−0.09(0.90)	−0.09(0.90)
	Bars-Rev	0.30	−0.002 (−0.01)	0.08 (0.27)	0.26 (0.87)	0.27 (0.90)	0.29 (0.97)	0.29 (0.97)
	iAF1260b	−0.30	−0.03 (0.10)	−0.16 (0.53)	−0.22 (0.73)	−0.26 (0.87)	−0.28 (0.93)	−0.28 (0.93)
	iJO1366	−0.29	−0.03 (0.10)	−0.14 (0.48)	−0.23 (0.79)	−0.25 (0.86)	−0.27 (0.93)	−0.28 (0.97)
Clustering coefficient (μ)	Algebra	0.80	0.66 (0.83)	0.75 (0.93)	0.76 (0.95)	0.79 (0.99)	0.80 (1.00)	0.80 (1.00)
	Bars-Rev	0.58	0.28 (0.48)	0.44 (0.76)	0.49 (0.84)	0.57 (0.98)	0.58 (1.00)	0.58 (1.00)
	iAF1260b	0.55	0.46 (0.84)	0.54 (0.98)	0.55 (1.00)	0.55 (1.00)	0.55 (1.00)	0.55 (1.00)
	iJO1366	0.58	0.45 (0.77)	0.55 (0.95)	0.57 (0.98)	0.57 (0.98)	0.57 (0.98)	0.58 (0.98)
Average Neighbor Degree (μ)	Algebra	44.35	22.88 (0.52)	38.15 (0.86)	42.12 (0.95)	44.13 (0.99)	44.25 (0.99)	44.35 (1.00)
	Bars-Rev	13.28	8.94 (0.67)	12.32 (0.93)	11.95 (0.90)	12.29 (0.93)	13.28 (1.00)	13.28 (1.00)
	iAF1260b	141.425	25.66 (0.18)	37.21 (0.26)	135.27 (0.96)	139.49 (0.99)	141.395 (1.00)	141.425 (1.00)
	iJO1366	159.46	30.85 (0.19)	150.43 (0.94)	42.79 (0.27)	157.45 (0.99)	157.79 (0.99)	159.46 (1.00)

**Table 3:** The recovery number of each dataset under β=0.1.

	Origin	H0k	H1k-HED	H1k-HD	HD-HED	H2K	H2.25k
Algebra	0.15	0.81	0.42	0.45	0.41	0.36	0.39
Bars-Rev	0.43	0.83	0.55	0.54	0.49	0.49	0.51
iAF1260b	0.04	0.20	0.04	0.04	0.05	0.04	0.05
iJO1366	0.29	0.65	0.31	0.28	0.27	0.29	0.30

## Data Availability

The network data that support the findings of this study are available through https://github.com/ilyaamburg/fair-clustering-for-diverse-and-experienced-groups (accessed on 27 September 2023) and https://www.cs.cornell.edu/~arb/data/ (accessed on 27 September 2023).

## Supplementary Materials

The following supporting information can be downloaded at: https://www.mdpi.com/article/10.3390/e25101390/s1, Figure S1: Statistical indices of network Algebra and its hull models; Figure S2: Statistical indices of network Bars-Rev and its hull models; Figure S3: Statistical indices of network iJO1366 and its hull models; Figure S4: The SIR epidemic contagion under 2% initial infected nodes on four datasets with different infection rate β; Figure S5: The SIR epidemic contagion under 5% initial infected nodes on four datasets with different infection rate β; Table S1: Notations used in this paper; Table S2: The recovery proportion of each dataset under β=0.3; Table S3: The recovery proportion of each dataset under β=0.5.
