# Supplementary material for: Hyper-Null Models and Their Applications
Source: Entropy (Basel). 2023 Sep 28;25(10):1390. doi: 10.3390/e25101390 (PMC10606013; doi:10.3390/e25101390)
Supplement: Supplementary file 1 [file entropy-25-01390-s001.zip › entropy-2625595-supplementary.pdf]

Supplementary Material

1. Table of Notations

The definition of some notations used in this paper are summarized in table S1.

Table S1: Notations used in this paper.

Notations	Description
$\mathcal{H}$	A hypergraph
V	The node set of $\mathcal{H}$
E	The hyperedge set of $\mathcal{H}$
n	The number of nodes in $\mathcal{H}$
m	The number of hyperedges in $\mathcal{H}$
C	The incidence matrix of $\mathcal{H}$
A	The adjacency matrix of $\mathcal{H}$
D	The hyperdegree matrix of $\mathcal{H}$
B	The hyperedge adjacency matrix of $\mathcal{H}$
D_e	The hyperedge degree matrix of $\mathcal{H}$

2. More experiments results

This section contains the extra result of the experiment, which contains the statistical indicators of the networks and the epidemic spreading result under various initial infected nodes.

2.1. Statistics

The results of statistics of three datasets Algebra, Bars-Rev and iJO1366 are shown in figure S1, figure S2 and figure S3.

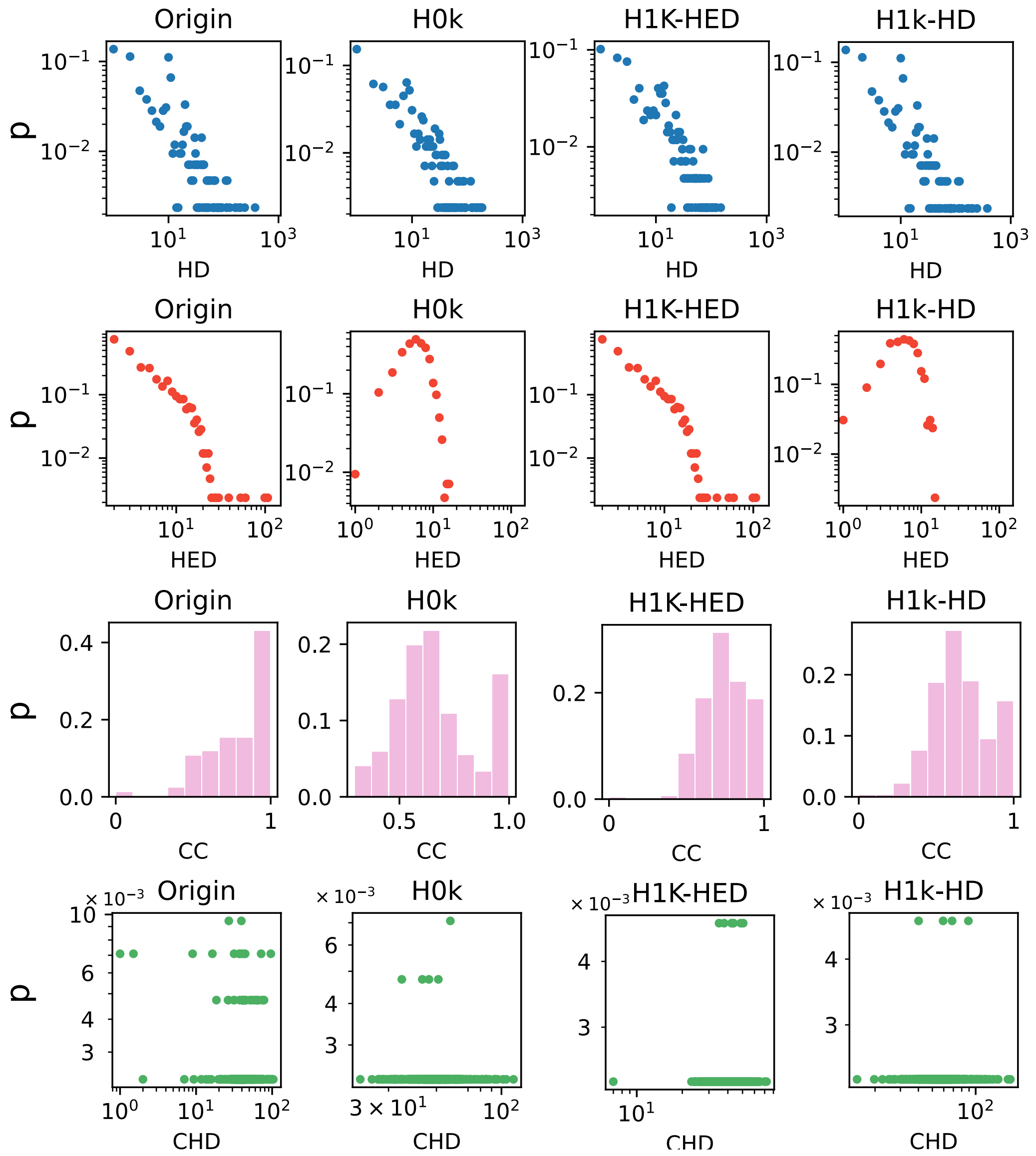

Figure S1: **Statistical indices of network Algebra and its hull models.** The x-axis in four rows from top to bottom represents the distribution of hyperdegree (HD), hyperedge degree (HED), clustering coefficient (CC) and co-average hyperdegree (CHD).

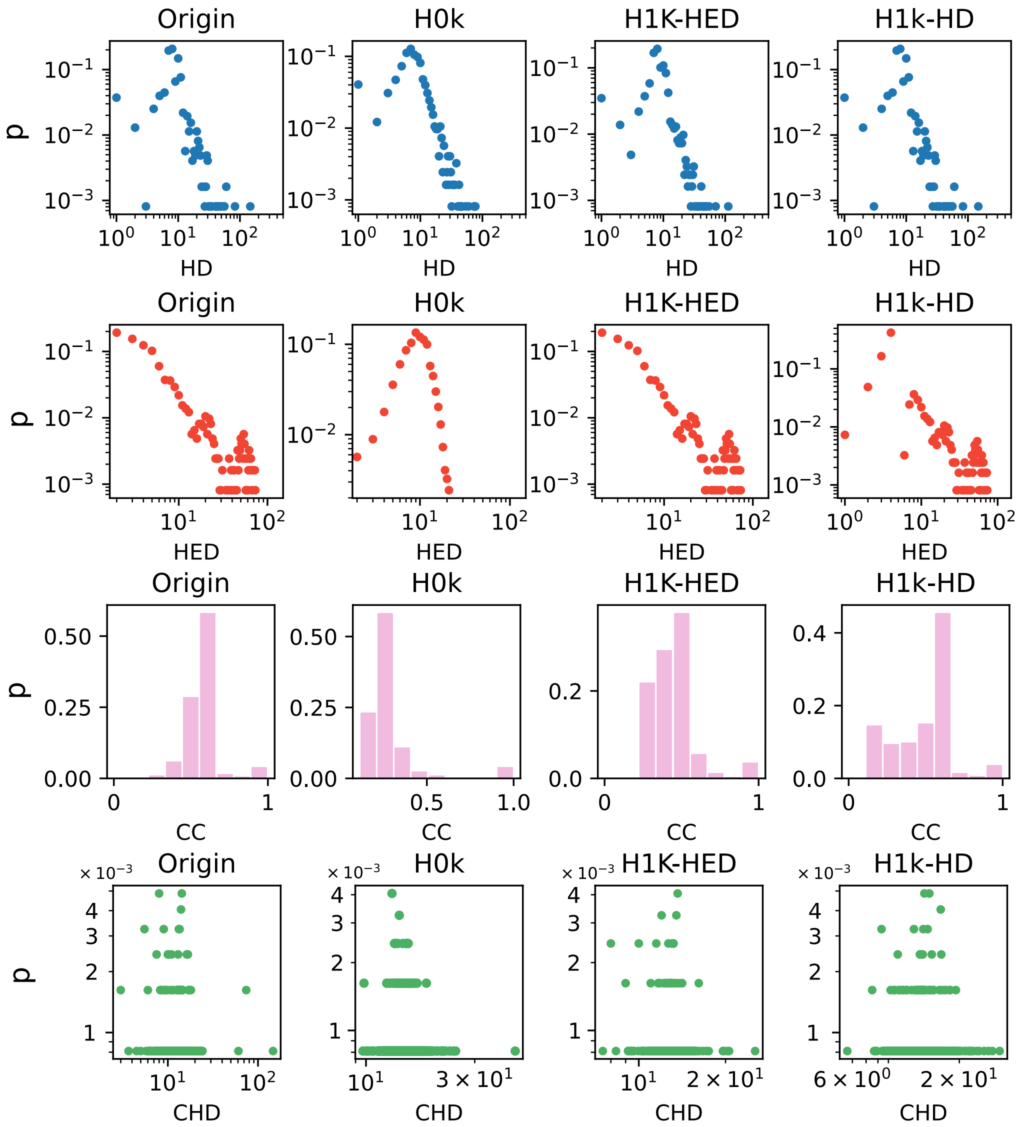

Figure S2: **Statistical indices of network Bars-Rev and its hull models.** The x-axis in four rows from top to bottom represents the distribution of hyperdegree (HD), hyperedge degree (HED), clustering coefficient (CC) and co-average hyperdegree (CHD).

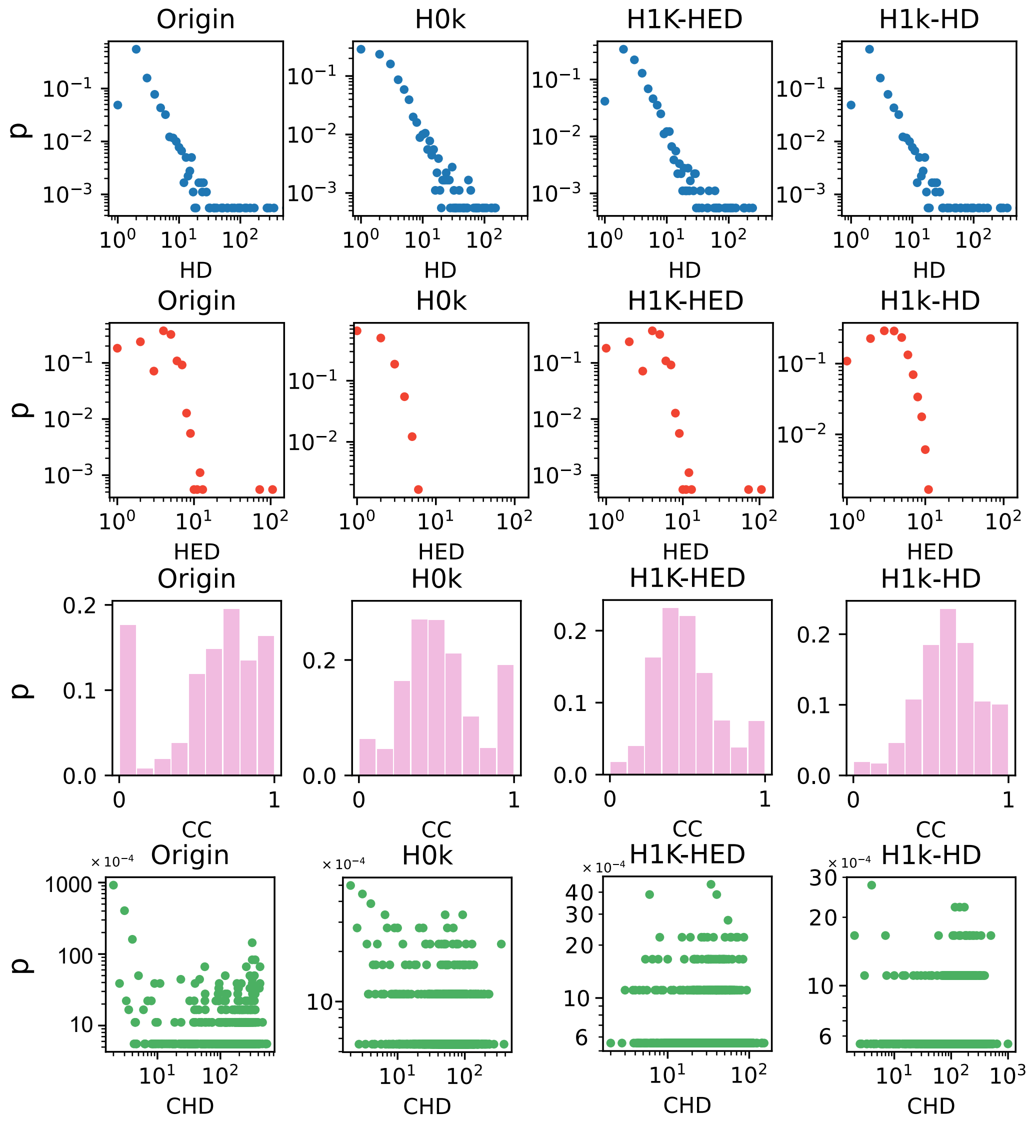

Figure S3: **Statistical indices of network iJO1366 and its hull models.** The x-axis in four rows from top to bottom represents the distribution of hyperdegree (HD), hyperedge degree (HED), clustering coefficient (CC) and co-average hyperdegree (CHD).

2.2. Epidemic Spreading

These section contains the results of epidemic spreading under 2% initial infected nodes in figure S4 and 5% initial infected nodes in figure S5.

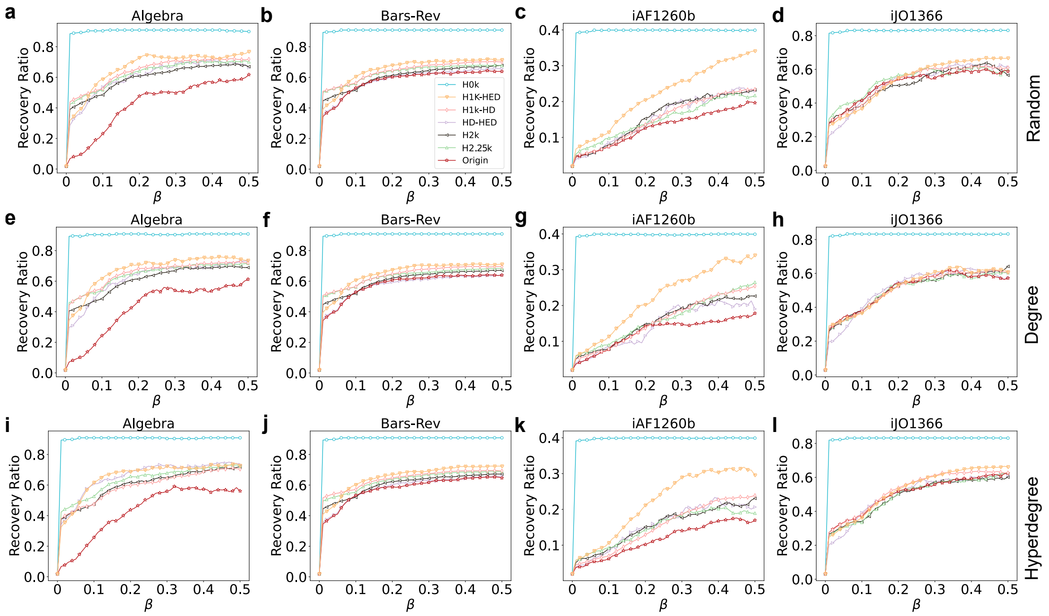

Figure S4: **The SIR epidemic contagion under 2% initial infected nodes on four datasets with different infection rate β.** The first (a-d), second (e-h), and third (i-l) row denotes the epidemic results on 2% initial infected nodes picked by random, degree, hyperdegree. The x-axis represents the different infection rates β and the y-axis denotes the recovery number ratio at the steady state.

Table S2: The recovery proportion of each dataset under $\beta = 0.3$

	Origin	H0k	H1k-HED	H1k-HD	HD-HED	H2K	H2.5k
Algebra	0.40	0.83	0.63	0.58	0.55	0.51	0.53
Bars-Rev	0.55	0.83	0.64	0.61	0.59	0.58	0.56
iAF1260b	0.08	0.20	0.12	0.09	0.09	0.09	0.12
iJO1366	0.43	0.66	0.42	0.44	0.44	0.46	0.45

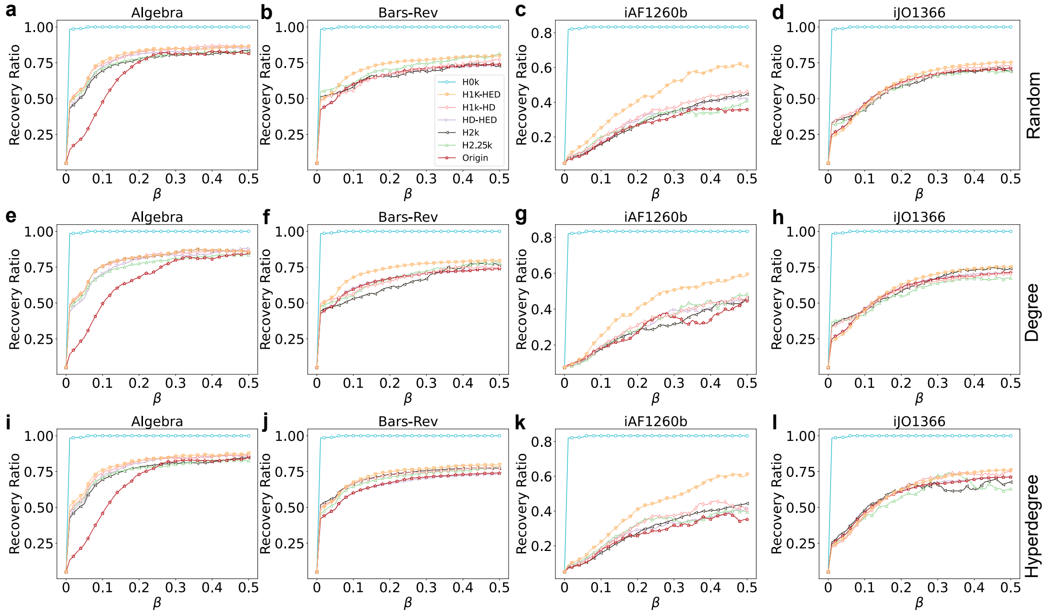

Figure S5: **The SIR epidemic contagion under 5% initial infected nodes on four datasets with different infection rate β.** The first (a-d), second (e-h), and third (i-l) row denotes the epidemic results on 5% initial infected nodes picked by random, degree, hyperdegree. The x-axis represents the different infection rates β and the y-axis denotes the recovery number ratio at the steady state.

In order to explore the spread of different datasets at the same infection rate, we counted the proportion of R state nodes when reaching the steady state in the case of $\beta = 0.3$ in table S2 and $\beta = 0.5$ in table S3.

Table S3: The recovery proportion of each dataset under $\beta = 0.5$

	Origin	H0k	H1k-HED	H1k-HD	HD-HED	H2K	H2.5k
Algebra	0.43	0.83	0.62	0.62	0.58	0.55	0.61
Bars-Rev	0.60	0.83	0.65	0.63	0.59	0.62	0.60
iAF1260b	0.12	0.20	0.15	0.13	0.13	0.11	0.12
iJO1366	0.47	0.66	0.52	0.50	0.47	0.50	0.45
